# Supplementary material for: Network analysis of toxin production in Clostridioides difficile identifies key metabolic dependencies
Source: PLoS Comput Biol. 2023 Apr 26;19(4):e1011076. doi: 10.1371/journal.pcbi.1011076 (PMC10166488; doi:10.1371/journal.pcbi.1011076)
Supplement: S1 Table — BHIS(G): Brain-Heart Infusion Supplemented (Glucose), Cd: C. difficile, CDMM: C. difficile Minimal Media, DCA: Deoxycholate, DMSO: Dimethyl Sulfoxide, GEO ID: Gene Expression Omnibus Identifier, TY: Tryptone Yeast. Alternate identifiers for RIPTiDe models with similar growth conditions are indicated in parentheses in the Growth Condition column when applicable; these identifiers are used for all analyses. (DOCX) [file pcbi.1011076.s001.docx]

| **GEO ID** | **Strain** | **Growth Condition (RIPTiDe Name)** | **Replicates** | **Contributors** | **Year** |
| --- | --- | --- | --- | --- | --- |
| GSE173804 | 630 | BHIS – Anaerobic (BHIS-1) | 3 | Weiss A, et al. [1] | 2021 |
|  |  | BHIS – microaerobic | 3 |  |  |
| GSE120189 | 630 | CDMM + low iron (Low Iron) | 3 | Berges M, et al. [2] | 2018 |
|  |  | CDMM + high iron (High Iron) | 3 |  |  |
| GSE165116 | 630 | BHIS (BHIS-2) | 3 | Tremblay Y, Monot M [3] | 2021 |
|  |  | BHIS + 120 uM DCA | 3 |  |  |
|  |  | BHIS + 240 uM DCA | 3 |  |  |
| GSE135912 | R20291 | BHIS (BHIS-3) | 3 | Lopez CA, et al. [4] | 2019 |
|  |  | BHIS + Calprotectin (Calprotectin) | 3 |  |  |
| GSE199109 | R20291 | BHIS + DMSO (DMSO-1) | 3 | Hurdle J, et al. [5] | 2022 |
|  |  | BHIS + Enoxolone (Enoxolone) | 3 |  |  |
| GSE107961 | R20291 | TY | 2 | Gu H, et al. [6] | 2018 |
|  |  | TY + 5 mM Cysteine | 2 |  |  |
| GSE86152 | R20291 | 5 mM DMSO (DMSO-1) | 4 | Sorg J, Monot M  [7] | 2019 |
|  |  | 0.5 mM DCA (Deoxycholate) | 4 |  |  |
|  |  | 5 mM Cholate (Cholate) | 4 |  |  |

**References**

1. Weiss A, Lopez CA, Beavers WN, Rodriguez J, Skaar EP. Clostridioides difficile strain-dependent and strain-independent adaptations to a microaerobic environment. Microb Genom. 2021 Dec;7(12):000738.

2. Berges M, Michel AM, Lassek C, Nuss AM, Beckstette M, Dersch P, et al. Iron Regulation in Clostridioides difficile. Front Microbiol. 2018;9:3183.

3. Tremblay Y, Monot M. Metabolic adaption to extracellular pyruvate triggers biofilm formation in Clostridioides difficile; 2021 [cited 2023 Apr 19]. Database: GEO [Internet]. Available from: <https://www.ncbi.nlm.nih.gov/geo/query/acc.cgi?acc=GSE165116>

4. Lopez CA, Beavers WN, Weiss A, Knippel RJ, Zackular JP, Chazin W, et al. The Immune Protein Calprotectin Impacts Clostridioides difficile Metabolism through Zinc Limitation. mBio. 2019 Nov 19;10(6):e02289-19.

5. Hurdle J, Marreddy R, Lee R, Phelps G. The licorice metabolite enoxolone attenuates Clostridioides difficile pathophysiology by corrupting its metabolic and toxin production networks; 2022 [cited 2023 Apr 19]. Database: GEO [Internet]. Available from: <https://www.ncbi.nlm.nih.gov/geo/query/acc.cgi?acc=GSE199109>

6. Gu H, Shi K, Liao Z, Qi H, Chen S, Wang H, et al. Time-resolved transcriptome analysis of Clostridium difficile R20291 response to cysteine. Microbiological Research. 2018 Oct;215:114–25.

7. Sorg J, Monot M. Effect of Bile Acids on C. difficile Growth; 2019 [cited 2023 Apr 19]. Database: GEO [Internet]. Available from: <https://www.ncbi.nlm.nih.gov/geo/query/acc.cgi?acc=GSE86152>
