# Supplementary figures and images for: Network analysis of toxin production in Clostridioides difficile identifies key metabolic dependencies
Source: PLoS Comput Biol. 2023 Apr 26;19(4):e1011076. doi: 10.1371/journal.pcbi.1011076 (PMC10166488; doi:10.1371/journal.pcbi.1011076)

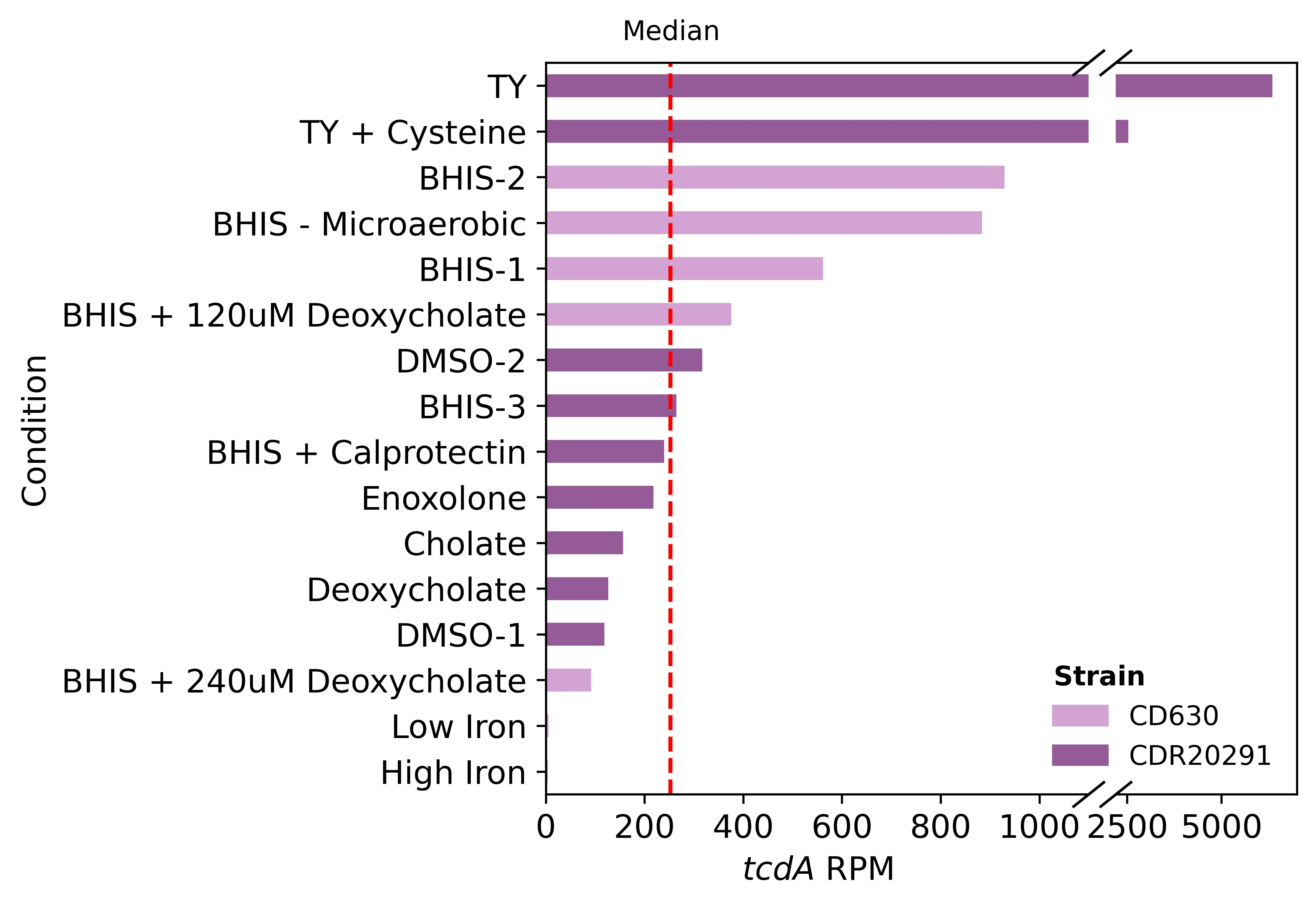

Supplement: S1 Fig — Toxin transcript counts quantified as reads per million (RPM) are shown for all conditions included in the study (see S1 Table for more details). Conditions were binned based on median tcdA transcript levels across all conditions and labeled as low (< median) or high (> median). (TIFF) [file pcbi.1011076.s002.tiff]

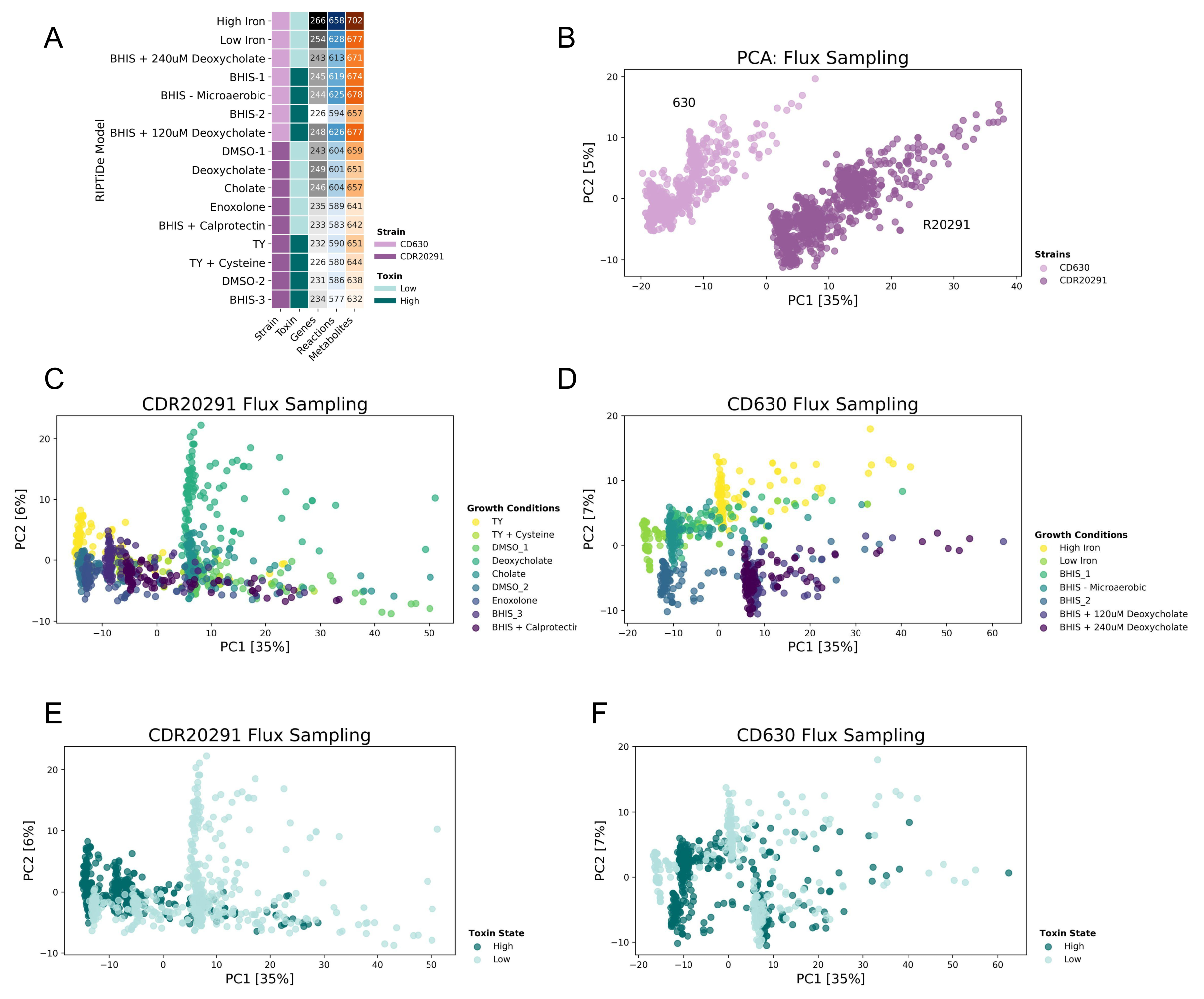

Supplement: S2 Fig — (A) Summary table of the RIPTiDe-contextualized models including the strain, toxin production level, and number of genes, reactions, and metabolites. (B) The iCdG709 (CD630, light purple) and iCdR703 (CDR20291, dark purple) C. difficile models were contextualized with transcriptomic data (S1 Table) and flux distributions were sampled (n = 500) using RIPTiDe. The flux sampling for each model was randomly down-sampled to 100 flux samples and PCA was performed for all the models together (B) and by strain (C–F). (TIFF) [file pcbi.1011076.s003.tiff]

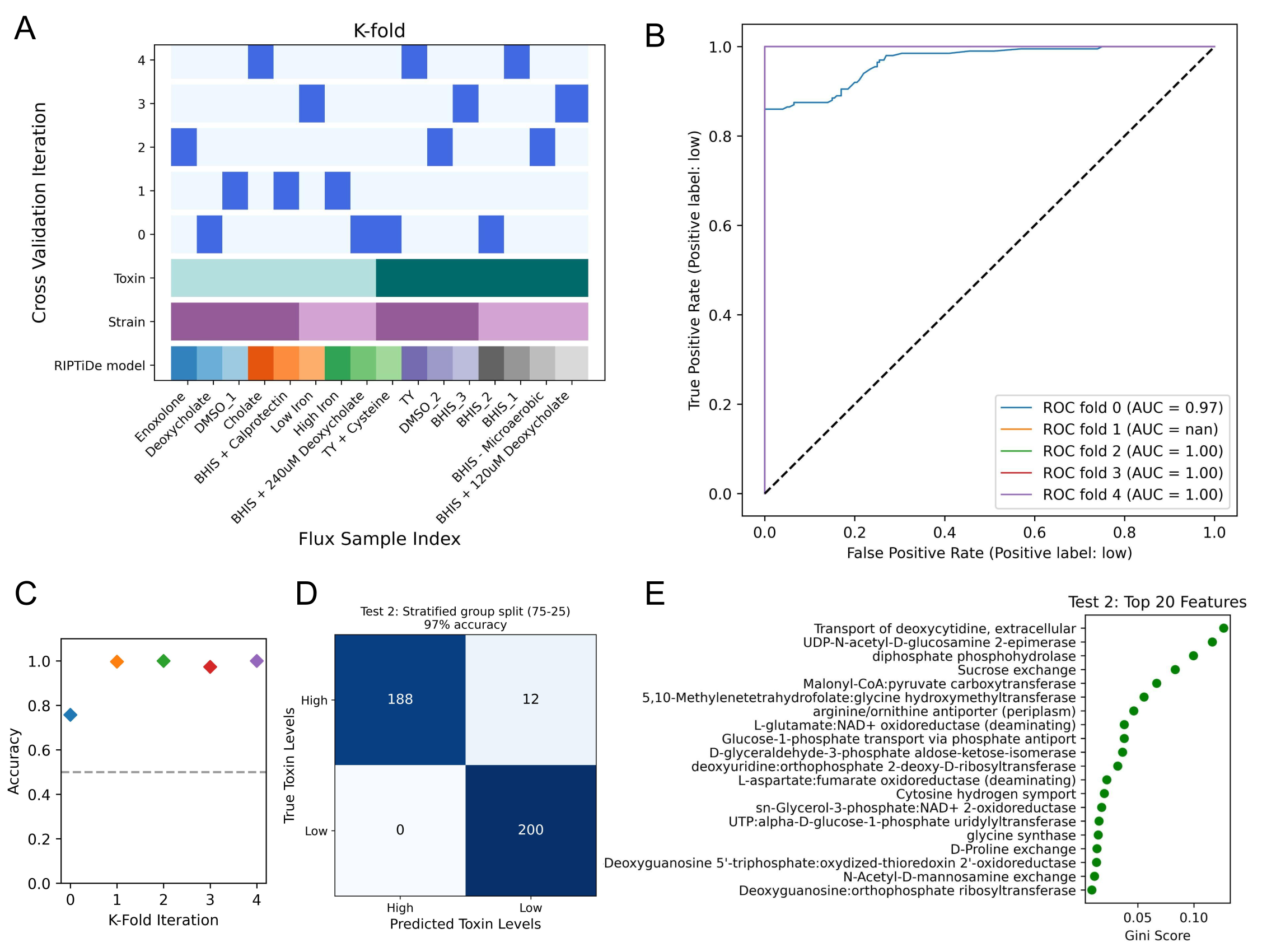

Supplement: S3 Fig — (A) Visualization of the random stratified group k-fold splits used for cross validation of the Random Forest classifier. (B-C) K-fold cross validation (k = 5) of the Random Forest classifier testing ROC (B) and accuracy (C), with an average accuracy of 95% in cross validation. (D) Confusion matrix for model predictions with train and test sets selected in a 75–25 ratio using random stratified group splits. The model trained on this set had a 97% accuracy. (E) The top 20 features for model predictions by Gini score. (TIFF) [file pcbi.1011076.s004.tiff]

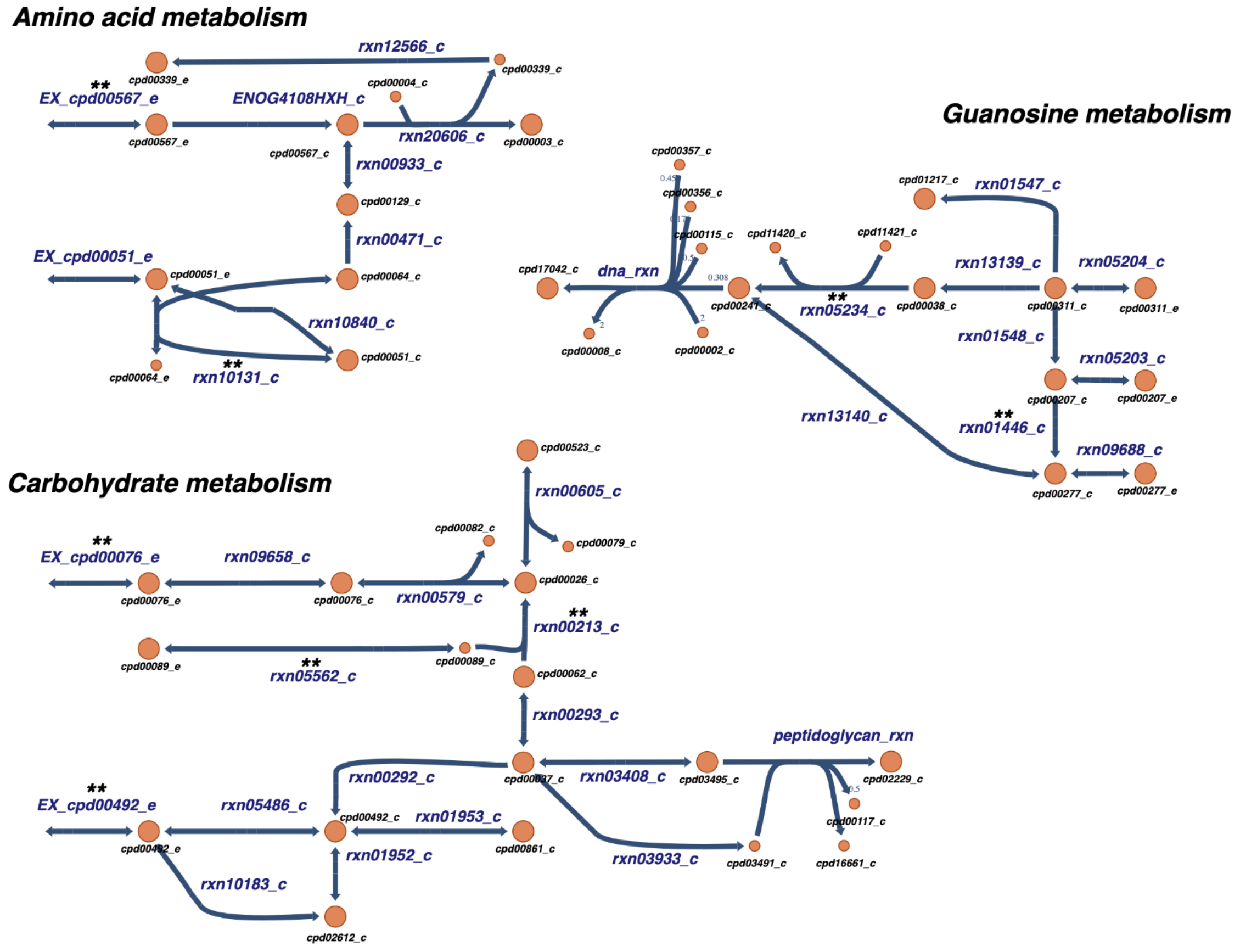

Supplement: S4 Fig — Metabolic context for reactions from the Random Forest analysis labeled with the reaction and model IDs from the GENREs iCdG709 and iCdR703. (TIFF) [file pcbi.1011076.s005.tiff]

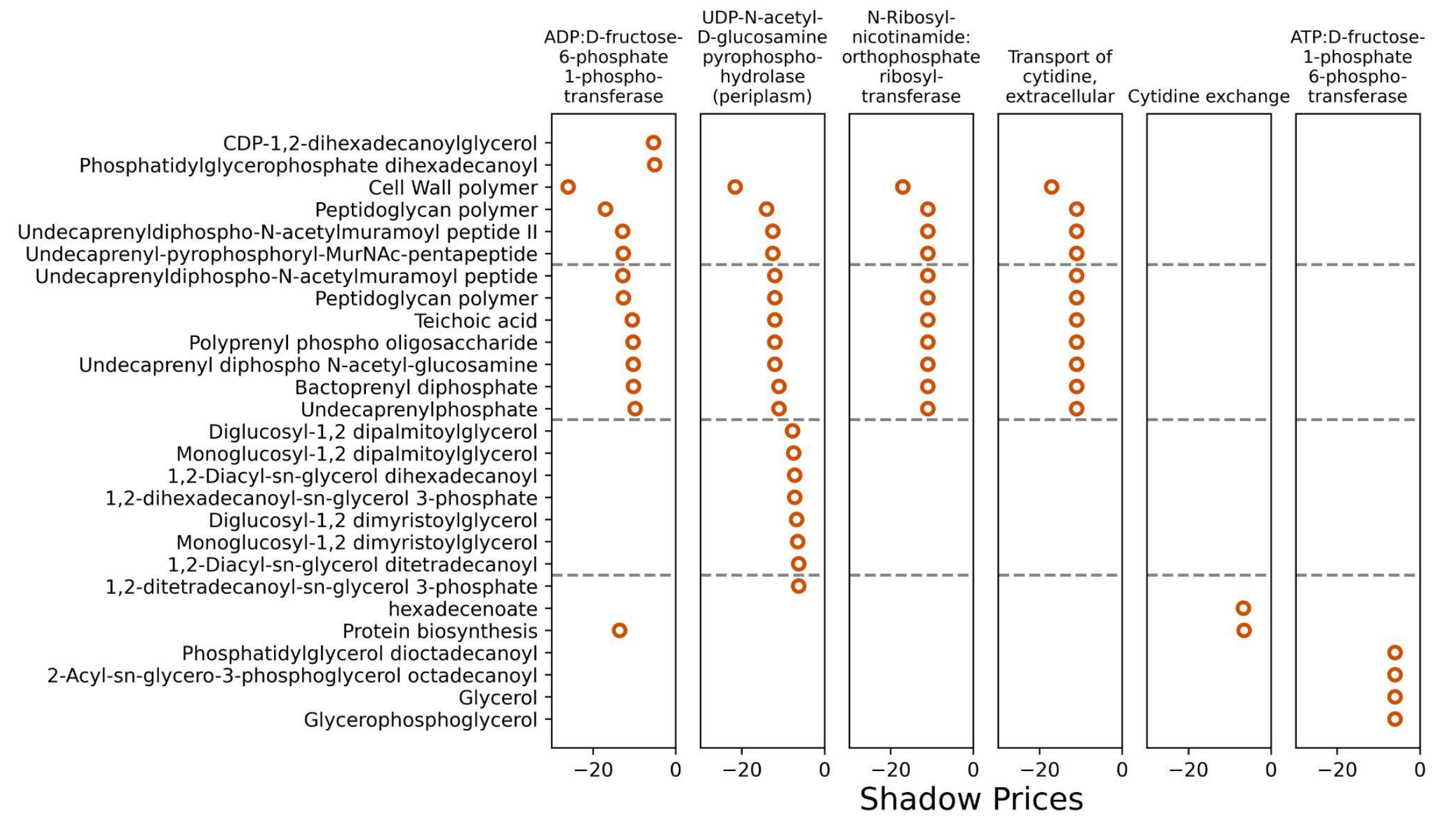

Supplement: S5 Fig — For each objective function (OF) listed in Fig 3A, the metabolites categorized as decreasing and with a shadow price < -5 are shown. (TIFF) [file pcbi.1011076.s006.tiff]

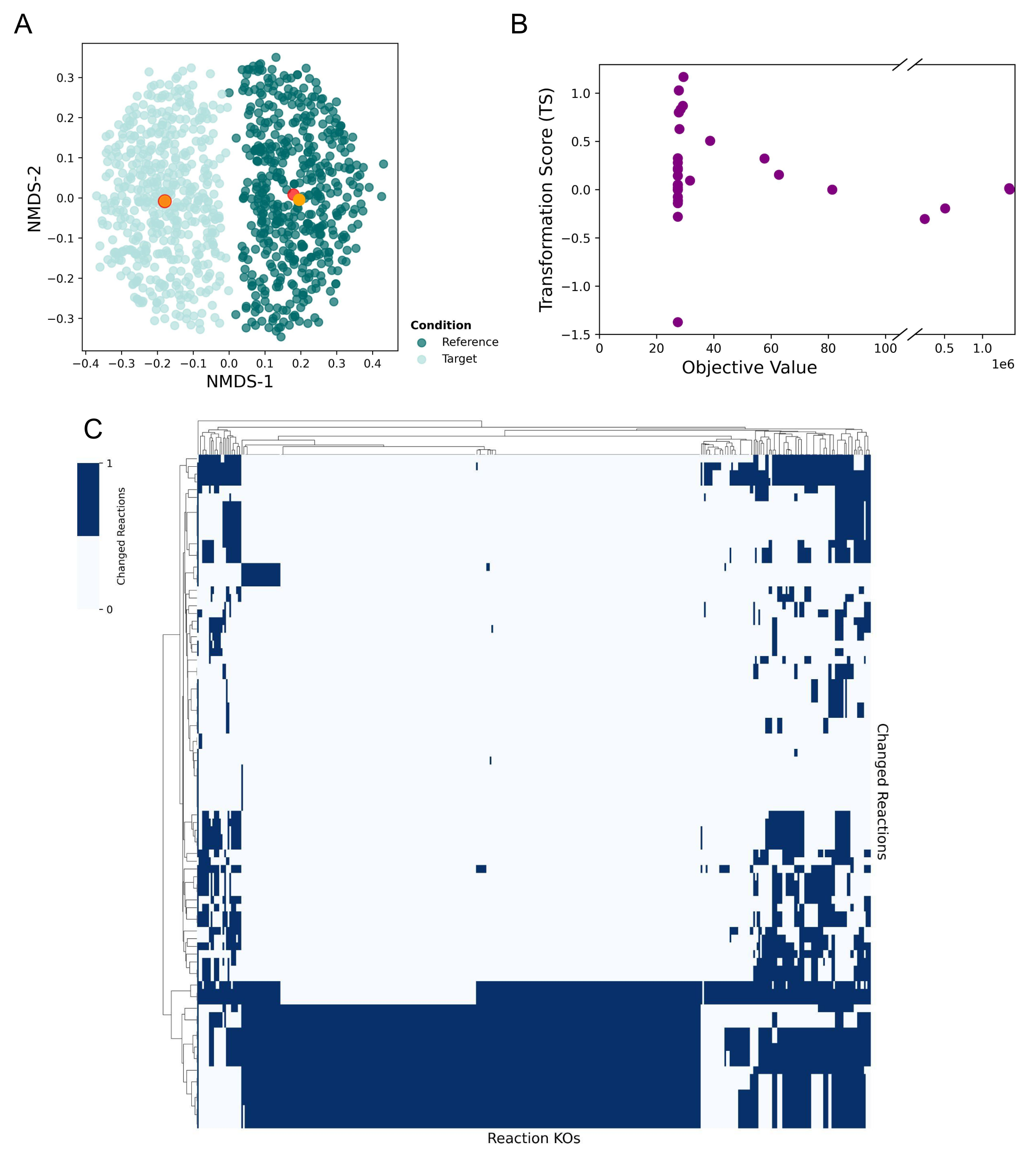

Supplement: S6 Fig — (A) Bray-Curtis NMDS of flux sampling results for iCdG709 contextualized for BHIS + DCA 240 uM (target, low toxin, light teal) and BHIS (reference, high toxin, dark teal) was used to calculate the centroids (red) and the flux sample closest to the centroid (orange) for each model. (B) The MIQP objective value verses the TS demonstrates the utility of the TS in ranking flux solutions with a similar objective-value based on success of the flux solution in transforming reactions to the target state. (C) Successfully changed reactions for each reaction knockout. Successful (dark blue), unsuccessful (light blue). (TIFF) [file pcbi.1011076.s007.tiff]

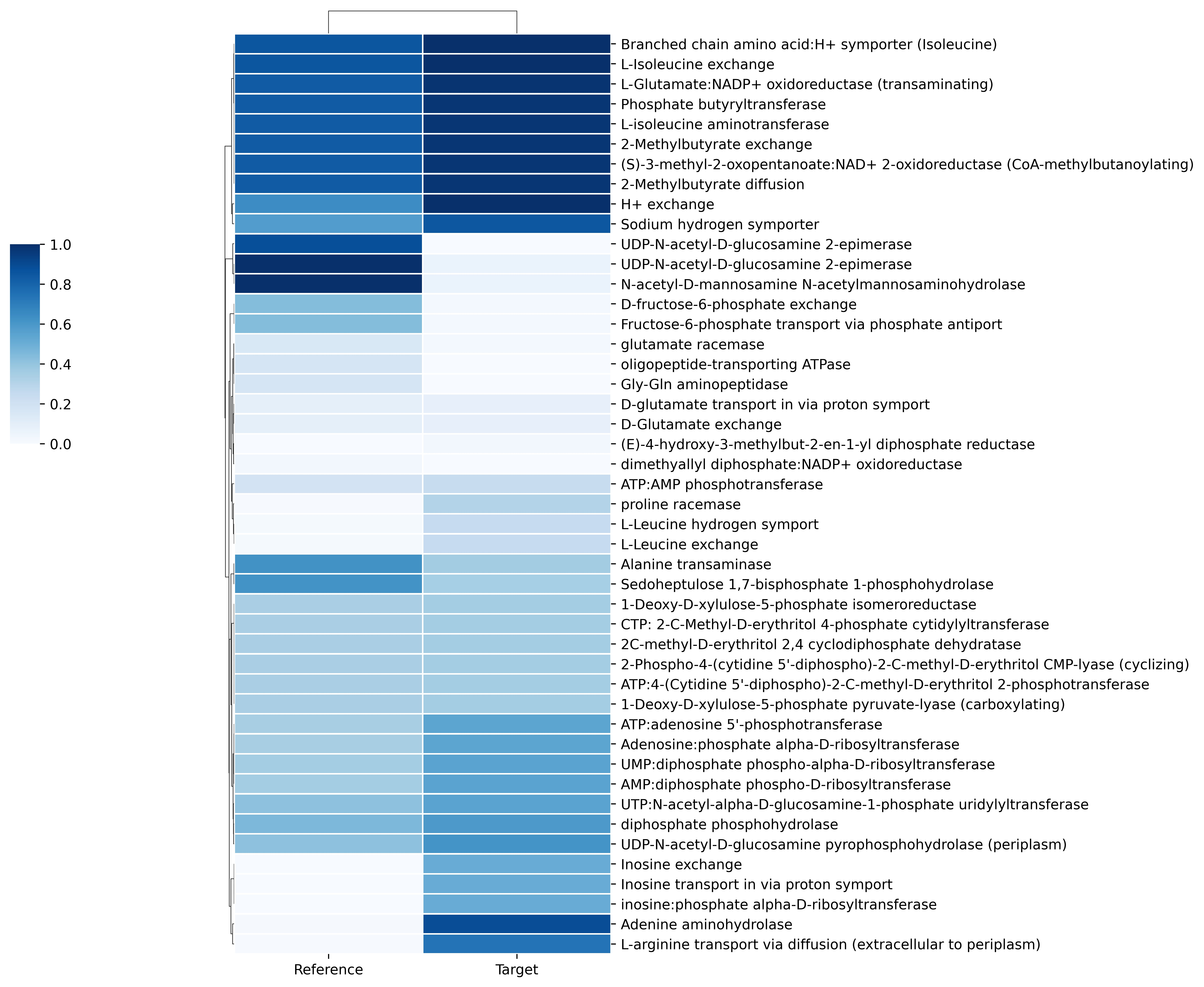

Supplement: S7 Fig — (TIFF) [file pcbi.1011076.s008.tiff]

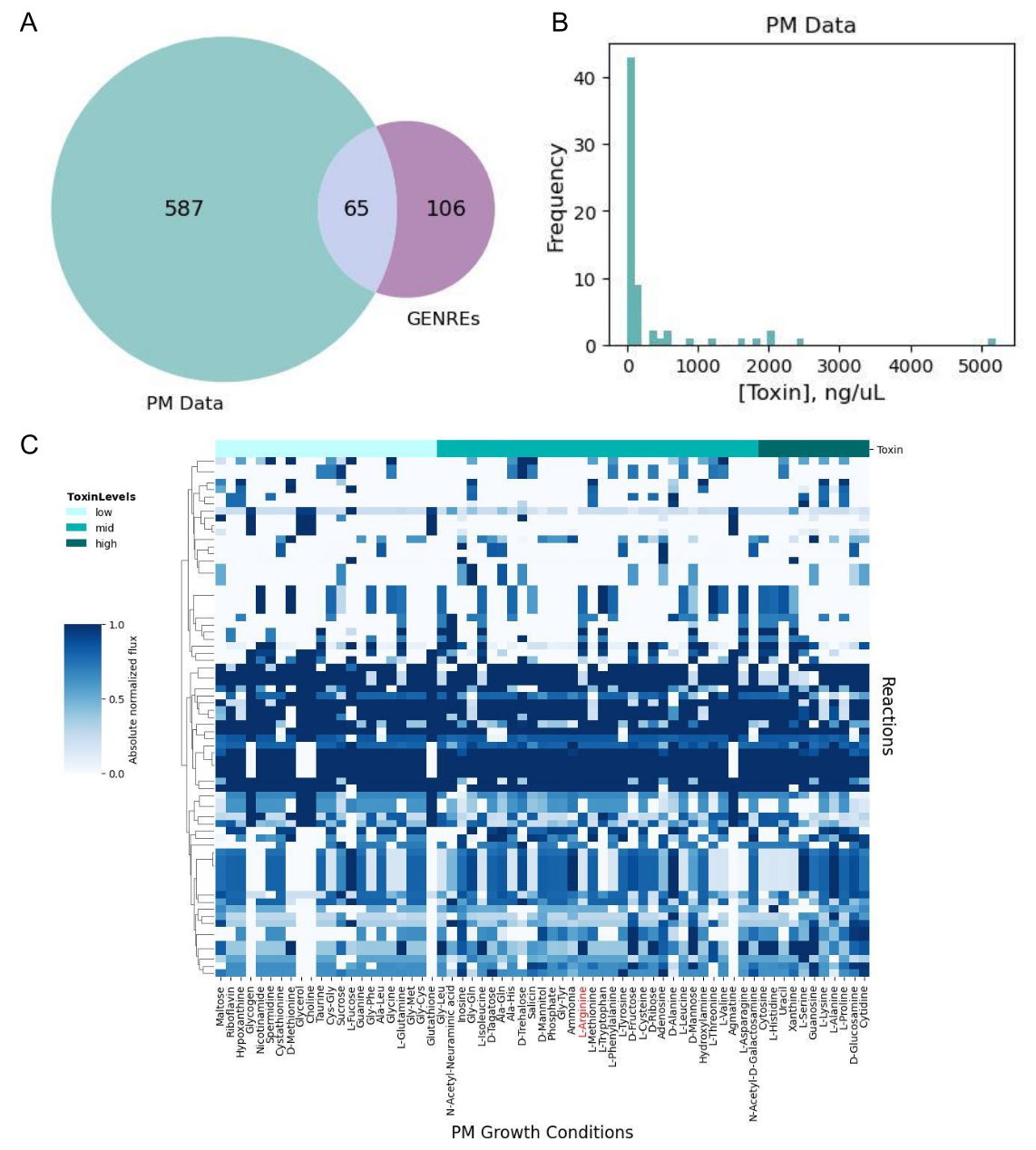

Supplement: S8 Fig — (A) Venn diagram showing the overlap of unique metabolites from the PM dataset and the extracellular metabolites from the GENREs. (B) The toxin concentration distribution for the 65 overlapping growth conditions from panel (A). (C) Simulated reaction flux through each in silico PM condition (n = 65). The flux data was min-max normalized and reactions with flux variance across all conditions < 0.05 were removed and the absolute flux value of the remaining reactions was visualized. The PM growth conditions are sorted by their toxin category. Toxin categories were defined as low (<42 ng/uL), mid (42–420 ng/uL), and high (>420 ng/uL) as in Lei, XH and Bochner, BR (2013). (TIFF) [file pcbi.1011076.s009.tiff]
