# Supplementary material for: Network analysis of toxin production in Clostridioides difficile identifies key metabolic dependencies
Source: PLoS Comput Biol. 2023 Apr 26;19(4):e1011076. doi: 10.1371/journal.pcbi.1011076 (PMC10166488; doi:10.1371/journal.pcbi.1011076)
Supplement: Attachment — Submitted filename: PLOSCompReviews.docx [file pcbi.1011076.s013.docx]

We appreciate the thoughtful reviews which have helped improve the manuscript significantly. Below are original reviewer comments in **black** and our responses in **blue.**

**------------------------**

**Reviewer #1:**

The authors present a computational analysis of metabolic regulation of toxin (TcdA and TcdB) production in *C. difficile*. They integrate public transcriptomics data to develop 16 context specific metabolic models, using the RIPTiDe algorithm and *C. difficile* metabolic models. These base models and algorithm were previously developed by several of the same authors. They analyze the models using a combination of flux sampling, machine learning (random forest), and shadow prices to identify patterns associated with toxin production. They also implement the metabolic transformation algorithm to identify reaction knockouts that drive metabolic flux towards a low-toxin state. The discussion of the results focuses on arginine and ornithine transporters and isoleucine. Overall, the scope and motivation of the work is interesting. The conceptual model is set up well. The analysis and interpretation of the results should be explained more clearly. Specific major and minor comments are provided below.

Major Comments:

1. I am concerned with overfitting in the machine learning results. The total number of samples is quite small (16). I understand that there are additional samples created through the flux sampling process, but these will likely be highly correlated. The model can likely learn, from these correlated samples, which condition a flux sample comes from and use that information to infer the toxin level. Therefore, the degree to which the results correspond to microbial physiology in toxin production vs condition specific physiology is not clear. This should be addressed in the discussion.

This is an excellent comment. We have redone the analysis, incorporating all of the suggestions for improvement made below (random stratified group sampling, feature selection using near-zero variance and Pearson’s correlation). We extended the discussion of the machine learning analysis to address limitations and how they impact interpretation of results (Lines 281-293).

2. To further address overfitting to conditions in the results, I would also suggest that the authors implement a cross-validation scheme where flux samples from the same condition are not mixed across the train and validate sets. To do this you can randomly select a set of conditions (of the 16) to assign to the training set (repeating random selections for statistics). The random selection could be stratified to ensure there are always some high and some low toxin samples. Then use all the flux samples from the randomly selected conditions in the training set and the remaining samples in the validate sets. Cross-validating in this way will give insight into whether the ML model can generalize across conditions and should be presented alongside the current results.

Thank you for this helpful suggestion. We have since implemented a random stratified group k-fold cross-validation where k=5. In this revised cross-validation method an equal (or near equal) ratio of low and high toxin conditions are present in the train and test sets and all of the flux samples from a RIPTiDe model are included in either the train or test set. We split the flux sampling data used for model predictions using a similar random stratified group splitting with a 75-25 train-test ratio. The updated methods and results are described in greater detail in the text (Lines 345-353 and Lines 113-117).

3. Another suggestion for the machine learning analysis is to reduce feature correlations. ML algorithms, and in particular feature importance calculations, are typically more stable when the input features are not correlated. Before implementing the ML the authors can clean up the input flux features in two ways. First, reduce the number of features by getting rid of any flux that has variability across all samples below some specified threshold (these features contain little to no information). Second, cluster features with covariance across all samples above some threshold into one feature (one of these fluxes can be used as a representative feature for the machine learning). There are often linear pathways of reactions with highly correlated flux across samples that can be reduced in this way. Reducing the feature space could make the results more stable and easier to interpret. If any feature engineering of this sort has already been implemented, the authors should include the details in the methods section.

Thank you so much for these detailed suggestions! We applied both of the recommendations for feature selection. We removed features with near-zero variance (NZV) < 0.005 and an absolute Pearson’s correlation coefficient > 0.8. Using these filters we were able to reduce the number of features from 482 to 100. These updates are detailed in the text (Lines 344-345).

4. The authors could further discuss the relationship between the ML analysis and the MTA analysis. What are the differences in the types of results you expect to find with these two approaches? How do the results from the two approaches complement or support each other?

We agree that the differences and complementarity of these analyses needed to be more clear. We updated the discussion to highlight these points (Lines 236-240).

Minor Comments:

1. Line 11. Consider changing “its toxins” to “two toxic proteins”. Defining TcdA,B more specifically as proteins (as opposed to metabolites) will help non-expert readers quickly understand the context of the modeling work.

We agree that this addition will enhance clarity and we have updated the text accordingly (Line 11).

2. Line 51-54. This is not a comment for this paper, but I was curious if the glucosylating activity of TcdA,B is a metabolic process that could be modeled. Is that included in the genome-scale metabolic models? Just a thought on another possibly interesting line of inquiry.

Great question; TcdA,B glucosylation involves complex cellular processes such as binding, signaling, endocytosis, and cleavage which generally fall outside the scope of metabolic models. TcdA,B protein synthesis is not included in our models of *C. difficile* for this reason. However, TcdA,B glucosylation could potentially be modeled using *in silico* molecular methods like quantitative structure-activity relationship (QSAR) models. Additionally, the review [*Clostridium difficile Toxin Biology*](https://pubmed.ncbi.nlm.nih.gov/28657883/) by K. Aktories, et al. provides an excellent illustration of the known mechanism of TcdA,B glucosylating activity. We added this as an idea for future directions in the discussion (Lines 317-318).

3. Line 81-83. It would be nice to include in the introduction a bit more description of the transcriptomic data that was used. Is this all from one study, multiple studies? Is there any motivation behind the different conditions that were included? Maybe just a reference to Supplemental Table 1 would suffice.

We first filtered RNAseq studies based on the strain (Cd630, CdR20291) because we have high quality reconstructions for these two strains; we further filtered by whether the data and associated metadata was accessible. Using this selection process, we were able to get a close to even split of conditions for the two strains Cd630 and CdR20291. We have updated text in the introduction to state the data is from multiple studies and have added a reference to Supplemental Table 1 (Lines 79-81).

4. Line 98-99. Is there any reason for choosing tcdA over tcdB? Are tcdA and tcdB correlated across conditions?

We opted to use TcdA as the measure for low/high toxin state because TcdB had low to no expression across all conditions with the exception of the Tryptone Yeast conditions (TY and TY + Cysteine) while TcdA expression had a more even distribution of expression across all conditions (Supplemental Figure 1). The expression levels of TcdA and TcdB were not correlated in the transcriptomic datasets we used for this study. We updated the methods section to articulate the reason for this choice (Lines 328-330).

5. Line 107-108. Please provide additional description of the flux sampling here or point to this description in the methods. Was this sampling of the entire feasible steady-state space, was biomass production optimized and used as a constraint?

We have included additional details for the flux sampling protocol in the methods section and have added a reference to the methods in the results section (Lines 338-341 and Lines 108-109).

6. Paragraph 111-121. The machine learning approach should be described in more detail. How many sampled flux distributions were used for each condition? What was the train/validate/test split procedure? What was the accuracy of the ML model on the test (or validation) set relative to a null distribution? How was feature importance extracted from the random forest model? This information is important for interpreting the results so it should be presented to some degree in the results section.

We agree that these details are needed to aid interpretation of the results. Accordingly, we have added additional details regarding the number of flux samples per condition, the train-test-split protocol, model accuracy, and evaluation/extraction of feature importance to the results section as well as referenced the methods section for further description (Lines 113-117 and Lines 345-351).

7. Line 122. Figure 2. It may be nice to highlight the arginine and ornithine transport related reactions. The figure in part B does a nice job of explaining the context of the reactions from the ML importance results in part A. Maybe highlight the relevant reactions in A with bold or a different colored font and link them to the matching reaction in part B with a superscript.

We agree that this update will make it easier to interpret the figure. We highlighted the reactions from the Random Forest analysis (Fig 2B) that are contextualized in the flux map (Fig 2C) using asterisks. Additionally we bolded the reaction edges in 2C for reactions that are in 2B (Figure 2). The key for the interpretation of these highlights is explained in the figure caption.

8. Paragraph 130-141. It would be nice to have more discussion of why certain reactions have many sensitive metabolites while others do not. How could this arise and what are the implications? In general, what are the implications of a metabolite having a strong shadow price for an important reaction? I was surprised to see that arginine and ornithine do not come up in the shadow price analysis, as I would naively think that they would limit the transport reactions. The authors could expand on this in the results section here or in the discussion.

We have added additional details to the results section to clarify the implications of the shadow pricing results (Lines 138-147).

9. Line 145. The blue column seems to be a fraction of models not the number of models.

Thank you for noticing this; we have corrected the figure caption (Line 155).

10. Line 143. Figure 3b. It would be good to include the ID of the metabolites or some other more specific name.

We agree that model specific IDs for metabolites and reactions increase reproducibility. To retain human readability of the graphic we have not changed the format for Figure 3b, but we have included a version with model IDs in Supplemental Figure 4 for interested readers. We have additionally submitted the flux sampling, shadow pricing, and MTA data as Supplemental Data Files 1, 2, and 3 respectively.

11. Paragraph 155-177. The MTA section seems to be only weakly connected to the previous results from the ML section. Any efforts to link the two results in the discussion would be appreciated. (See major comment 4)

We have extended the discussion of the machine learning results in relation to the MTA results to strengthen the link between the two analyses (Lines 236-240).

12. Line 183. Include the value of epsilon in the caption.

Epsilon is a vector of unique thresholds used to determine if the vector of reaction flux changes are statistically significant. We agree that this is confusing and have removed it from the figure caption and reworded the definition of epsilon in the methods (Lines 412-415).

13. Paragraph 240-250. Good discussion of implications of the RIPTiDe algorithm. Additional discussion should be added regarding the limitations of GENRES and the other analyses utilized here.

We have included additional paragraphs discussing the limitations of flux sampling, machine learning, shadow pricing, and GENREs (Lines 281-293).

14. Line 455. Sup Fig S2. An additional PCA plot with high and low toxin as the colors would be good to include here.

This is a good suggestion. We have added PCA plots by strain colored by toxin state (Supplemental Figure S2, E-F).

15. Lines 488-489. A numbering system is mentioned in the figure caption, but I do not see any use of that numbering system in the table. Maybe it would be clearer to include the RIPTiDe model reference in the table.

We have updated Supplemental Table 1 with the alternate identifiers used for RIPTiDe models with similar growth conditions.

16. Line 348. The github repository that is linked seems to be empty at this time.

Thank you for making us aware of this omission. We have updated the GitHub repository with all of the relevant scripts and data (<https://github.com/dap5mb/cdToxinAnalysis>).

**Reviewer #2:**

This is a review of “Network analysis of toxin production in *Clostridioides difficile* identifies key metabolic dependencies” by Powers and colleagues. This paper focuses on *Clostridium difficile*, a notorious opportunistic pathogen which has a diverse metabolism that enables it to establish a niche within the complex gut environment. Its pathogenesis is primarily mediated by toxins TcdA and TcdB. This paper successfully demonstrates how toxic production is regulated by the organism's metabolism, a long-standing question in the field. The study employs a system biology-based workflow, utilizing genome-scale metabolic models, to reveal how different extracellular environments can affect the regulation of toxin production and how this relates to changes in intracellular metabolism.

This is a strong paper especially because of its innovative use of publicly available transcriptomic data from various studies to provide an extracellular context for the genome-scale metabolic models. The choice of low and high toxin states through transcriptomic data gives these states the context while performing metabolic modeling using genome scale metabolic models. The contextualization of these models has shown how these states are influenced by both extracellular and intracellular environments.

The use of the RIPTiDe algorithm and machine learning methods highlights the key role that arginine and ornithine, which are available from the environment, play in regulating toxin production. The paper goes on to show which is further regulated by intracellular pools of fatty acids and large polymer metabolite pools, as shown by the flux balance analysis and shadow pricing analysis. Additionally, the application of the mMTA algorithm identifies important reactions involved in transitioning from high to low toxin production states, providing ideas for potential therapeutic targets.

I am convinced that this paper will be a valuable contribution to the field. However, there are important issues that should be addressed before publication:

Major issues:

1. The paper states that the analysis code is shared but the associated link is empty. This needs to be fixed before publication.

Thank you for making us aware of this omission. We have updated the GitHub repository with all of the relevant scripts and data (<https://github.com/dap5mb/cdToxinAnalysis>).

2. The methods section could be more detailed in describing the usage and caveats of the RIPTiDE algorithm and shadow pricing analysis with respect to flux sampling to improve the reproducibility of the work.

We have updated the methods section with more specific details for the application of RIPTiDe for flux sampling and for the shadow pricing analysis (Lines 338-341 and Lines 364-373). For additional details regarding the usage of RIPTiDe we highly recommend the RIPTiDe repository which contains multiple usage examples (<https://github.com/mjenior/riptide>).

Minor issues:

3. The paper is a valuable contribution to the field's understanding of toxin regulation by metabolism in C. difficile. However, it would have been beneficial to include a discussion of isoleucine fermentation as a source of energy metabolism in the results section.

We agree that additional context is helpful here. We have added a brief discussion of isoleucine fermentation in the results and a more thorough discussion in the Discussion section (Lines 190-191 and Lines 254-267).

4. The abstract and introduction effectively summarize the work and highlight its potential therapeutic applications, but the results and discussion sections could benefit from more specific recommendations on this subject.

We agree that additional context could be beneficial. We contextualized our study in relation to therapeutic applications within the field in the discussion (Lines 309-315).

5. Additionally, the figure legends in Figure 3A could be clearer, and the use of the term "context dependent" for metabolites with shadow price>2 may be confusing.

Thank you for this insight. We have amended the figure legends and caption; specifically, we changed “Context Dependent” to “Variable” to reflect that the value of these metabolites’ shadow prices have significant variation across models (Figure 3A, Lines 158-159).

6. The legend in Figure 4 should also be more descriptive to make it accessible to non-experts in computational methods, and the use of the term epsilon without further explanation may be confusing to readers.

We agree that the figure caption was not very clear. We have removed the term epsilon and instead state that successful reactions are classified as those with changes in flux in the desired direction above a minimum threshold of significance and provide a more detailed explanation of epsilon in the Methods section (Lines 198-200 and Lines 412-415).

**Reviewer #3:**

This paper reports integration of publicly available transcriptomic data using RIPTiDe algorithm to create contextualize how nutritional changes regulate toxin levels in C. difficile. This work is built on previously published genome scale models of strains 630 and R20291. While it is interesting to see transcriptomic data integration into the previously published model, it is incremental work. The following aspects could make this paper much stronger.

1. Integrate actual toxin level data following growth in Biolog Phenotype array previously reported by Lei and Bochner 2013. That paper is cited as ref#26 in the discussion. However, that data is not integrated with the model. There is some agreement between the results in the current work and phenotypic toxin data published in ref#26. For example, Arg-dipeptides showed higher toxin production in Biolog plates. Calibrating the FBA model with validated phenotypic toxin data will make this work much better. Since authors have done that previously in E. coli (data guided FBA model), doing that here should not be that difficult.

Integration of Biolog data with metabolic models is a great way to associate environment-phenotype relationships with metabolism. Pairing transcriptomic data with the model using RIPTiDe is another way to associate environment-phenotype relationships with metabolism, where the transcriptomic data and experimental conditions can be considered as the environment and the toxin expression levels as the phenotype. Integrating Biolog data with the current analysis is not feasible as it has its own complete environment-phenotype relationship already associated. Therefore, with this revision we conducted a separate analysis using this data. Briefly, we used the reconstructions to simulate biomass production in a minimal media condition iteratively supplemented with a Biolog metabolite and measured flux. We processed the flux data using min-max normalization and filtered out reactions with variance < 0.05. We did a Pearson’s correlation test between this filtered flux dataset and the Biolog toxin measurements, but there were no correlations > 0.75. We also visualized the filtered dataset using a heat map which also did not show significant metabolic patterns by toxin level. This analysis can be found in greater detail in the manuscript (Lines 425-437, 218-220, Supplemental Figure 8).

2. The other question is how genome variation in C. difficile affects toxin production. CD 630 and CD R20291 belong to different toxinotypes. There have been conflicting reports including and excluding the importance of toxinotypes in C. difficile virulence. Although there is high genome variation in C. difficile, genes coding for the central metabolic pathways are somewhat conserved, albeit with some sequence variation. Is data integration with genome scale modeling sensitive enough to understand how the pathogenicity locus (where clusters of toxin genes are located) interacts with master regulators of central metabolic pathways (codY, CcpA, and others). Integrating Biolog data could be interesting because adenine and related compounds were the strongest toxin inducers in the previous study.

This is an excellent point. We have found genetic variation in *C. difficile* to have an effect on both metabolism and toxin production responses. In the machine learning analysis we did as reported in Figure 2, strain differences were a significant factor in predicting toxin transcript level. Integration of transcriptomic data with the model provides a contextualized picture of the metabolism of *C. difficile* under the genetic and environmental conditions associated with the transcriptomics data. However, the model is limited to metabolic genes, proteins, and metabolites and does not explicitly account for regulatory networks. Accounting for regulation in toxin production could be an interesting direction to expand on as we know that toxin responses are in part the effect of global regulators. We have added this as an idea for future directions in the discussion (Lines 315-320)

3. Public data set is available where both transcriptome and metabolome were analyzed following C. difficile infection in a mouse model (PMID: 29600278). Unfortunately, toxin levels were not reported in that paper. However, toxin gene expression level could be pulled from the transcriptome data, and do the predictions here correlate with what is reported in that work? I agree that dataset may not be amenable for modeling, but could be useful in expanding the discussion in the context of toxinotype variation.

Thank you for bringing up this interesting study. In selecting transcriptomics to include in our study, we selected publicly available *in vitro* studies of either CD630 or CDR20291 because we have well-curated metabolic models for these strains. In this cited study, the authors use *C. difficile* strain VPI 10463 in an *in vivo* experimental setting which is one reason we opted not to include it in our analysis. VPI 10463 is an incredibly powerful toxin producer and we expect the expression data for *tcdA* and *tcdB* to be quite high at the 12 and 24 hour timepoints. Though the authors have shared the raw transcriptomic data, they have not shared the count tables they used for the study which does not facilitate a direct analysis within the scope of our current study.
